# A review on initiatives for the management of daily medical emergencies prior to the arrival of emergency medical services

**DOI:** 10.1007/s10100-021-00769-y

**Published:** 2021-09-18

**Authors:** Niki Matinrad, Melanie Reuter-Oppermann

**Affiliations:** 1 Department of Science and Technology, Linköping University, Norrköping, 60174 Sweden; 2 Information Systems - Software and Digital Business Group, Technical University of Darmstadt, 64289 Darmstadt, Germany

**Keywords:** Emergency medical services (EMS), First responders, Volunteers, Community first responders (CFR), Automated external defibrillators (AEDs), Drones

## Abstract

Emergency services worldwide face increasing cost pressure that potentially limits their existing resources. In many countries, emergency services also face the issues of staff shortage–creating extra challenges and constraints, especially during crisis times such as the COVID-19 pandemic–as well as long distances to sparsely populated areas resulting in longer response times. To overcome these issues and potentially reduce consequences of daily (medical) emergencies, several countries, such as Sweden, Germany, and the Netherlands, have started initiatives using new types of human resources as well as equipment, which have not been part of the existing emergency systems before. These resources are employed in response to medical emergency cases if they can arrive earlier than emergency medical services (EMS). A good number of studies have investigated the use of these new types of resources in EMS systems, from medical, technical, and logistical perspectives as their study domains. Several review papers in the literature exist that focus on one or several of these new types of resources. However, to the best of our knowledge, no review paper that comprehensively considers all new types of resources in emergency medical response systems exists. We try to fill this gap by presenting a broad literature review of the studies focused on the different new types of resources, which are used prior to the arrival of EMS. Our objective is to present an application-based and methodological overview of these papers, to provide insights to this important field and to bring it to the attention of researchers as well as emergency managers and administrators.

## Introduction

Daily or everyday emergencies are frequent events with low magnitude of consequences (Quarantelli 1995). These emergencies can roughly be categorized into two groups of urgent and non-urgent. Within medical emergencies, urgent cases are life-threatening, and in case of a life-threatening emergency, such as an out-of-hospital cardiac arrest (OHCA) or a stroke, every second matters. While in case of a stroke a patient needs to be taken to the hospital as soon as possible to have a higher chance of survival, an immediate treatment at the scene is crucial for OHCA patients (Herlitz et al. 2005, 2003; Fothergill et al. 2013). Many countries have well-established and well-equipped emergency medical services (EMS) systems that send trained paramedics or emergency medical assistants to the scene of emergencies. However, almost all EMS systems worldwide face an increasing cost pressure, often accompanied by a shortage of staff and other necessary resources, as well as the issue of long distances to sparsely populated areas (Weinholt 2015; Yousefi Mojir and Pilemalm 2016). In many countries, the COVID-19 pandemic has exacerbated staff shortage and shown the importance of using all available resources as efficiently as possible. This means that adequate response times for all patients, 24 / 7, and throughout all regions during both normal and crisis times are difficult or even impossible to ensure.

Many planning alternatives for regular EMS exist that aim at minimizing response times while balancing the necessary and available resources. An overview on EMS logistics summarizing the planning problems and existing approaches as well as a description of a typical EMS system can be found in Reuter-Oppermann et al. (2017) or Bélanger et al. (2019), for example. Alternatively, to overcome the issues faced by the EMS and potentially reduce consequences of daily medical emergencies, several countries, including Sweden, Germany, the UK, and the Netherlands, have started initiatives utilizing new types of resources, human resources as well as equipment. Many initiatives send first responders or volunteers to the scene of emergencies to help patients before the EMS arrives. Others utilize automated external defibrillators (AEDs) that are located in public buildings or drones that can bring necessary resources to an emergency scene. These human resources and equipment form services that we call “pre-EMS services” in this study, because their utilization prior to the arrival of EMS can potentially contribute to saving lives of patients. Therefore, they can be described as services that help patients until the EMS arrives at the scene. While these services might help improve the response times, for example, they do not replace the regular system.

In this paper, we present a review of studies focused on pre-EMS services with the aim to provide insights to this field for both academics and practitioners. Even though the research field on pre-EMS services is relatively new, a good number of works exists that have studied the use of these new types of resources in medical emergency systems, from different perspectives including medical, technical, and logistical.

In this study, we focus on *pre-EMS services* and distinguish two main categories for them, (1) human resources (e.g., first responders and volunteers) and (2) equipment (i.e., AEDs and drones). With the aim to present a comprehensive overview on exiting literature related to pre-EMS services, we investigate the use of these services for the management of daily medical emergencies. These services can be used in response to bigger emergencies such as disasters as well. However, as the scope of this paper is daily medical emergencies, we exclude other types of events such as disasters or mass casualty incidents.

To the best of our knowledge, this is the first review targeting pre-EMS services. Rather than analyzing individual papers and their contributions to the field, we aim to give an application-based and methodological overview of these papers, to provide insights to this field and to bring it to the attention of researchers as well as emergency managers and administrators. We consider papers that cover both quantitative and qualitative methods from all operations research / operations management (OM) and medical journals and conference proceedings.

The remainder of the paper is structured as follows. First, we define our selection and classification scheme in Sect. 2 and describe the literature on pre-EMS services in Sect. 3. Then, in Sect. 4, we present an overview of the existing pre-EMS services in those countries that were named in the reviewed papers and it was stated that their initiatives are now operational. Based on the presented overview on human resources and on equipment in Sect. 3, we first present some insights for managers in Sect. 5 and then formulate directions for future studies in Sect. 6. We close the paper with a summary and conclusion in Sect. 7.

## Selection and classification scheme

The earliest published study within daily medical emergencies focused on one of the new types of resources that we found was from 1982. We limited the time period of our literature review to the end of 2020. We used Scopus as the main search engine and Google Scholar as the complementary one, and included publications from all journals and conference proceedings. We used “emergency medical services” in combination with each of the following words separately to find relevant papers: “first responders”, “automated external defibrillator”, “drone”, “unmanned aerial vehicle”, and “volunteer”. In addition, “stop the bleed” was searched individually. After several iterations of selection from all search results, which was initially 2127 papers that included duplicates as well, we selected 258 research papers that are included in this review. Besides duplicates, we excluded papers focusing on non-medical daily emergencies and disasters and on the application of the new types of resources outside of daily medical emergency context (e.g., physicians volunteering for studies conducted in a hospital). We also excluded papers that studied well-being (e.g., mental health) of new types of human resources or technological aspects of equipment (e.g., the technical design of drones or AEDs) as well as papers in which the focus was on EMS rather than pre-EMS services. On the medical side, we excluded papers focused on medical outcomes without any particular emphasize on pre-EMS services and their application within the studied medical emergency.

The included studies have used both qualitative and quantitative methods to investigate different problems such as cost-effectiveness of pre-EMS services, their deployment and task assignment, and location planning / placement of equipment. We included studies with a medical perspective in this review as well because we believe that it is insufficient for a comprehensive review to consider only operations research (OR) literature. Health services research, for example, investigates the impact of different care options. The results of these studies can be used as a basis and motivation for OR models that for instance aim to improve the access by locating care sites. In addition, medical studies already had a significant impact on OR models in the past. An example is the introduction of survival probabilities in ambulance location models. Our investigations showed that the majority of studies focused on human resources are conducted within the medical area. Overall, the medical studies included in this review mostly focus on ways of and benefits of incorporating equipment and human resources into the EMS systems, such as survival rates of patients or response times. Studies focusing on technical and logistical perspectives investigate questions such as the deployment of resources and the placement of equipment.

Based on the selected papers that are reviewed in this study, we identified four main categories, namely *type of emergency*, *type of data*, *methodology*, and *pre-EMS services*. The majority of researchers tend to focus on one type of emergency and pre-EMS services. Therefore, these two categories are important parameters that can describe an emergency scenario. Type of data and methodology are aspects that researchers consider once they have defined a problem, showing the solution scenario. They choose relevant methods to gather data and to perform required analysis, examining their hypothesis or testing their developed model. Therefore, we found these four categories as the most relevant aspects in providing insight into the pre-EMS services literature. We describe type of emergency, type of data, and methodology in this section and detail *pre-EMS services* in Sect. 3.

### Type of emergency

The majority of studies have considered one specific type of medical emergency. However, some works did not indicate a specific type of emergency or only excluded a specific type. Main recognized types of medical emergencies in the reviewed papers include OHCA, trauma, drowning, and bleeding. Emergencies of type trauma contain road traffic injuries as well. Papers that focus on bleeding are mostly concerned with the “stop the bleed” campaign and related studies. Consequently, we consider a total of five categories for emergency types: bleeding, drowning, general, OHCA, and trauma.

### Type of data

To investigate an identified problem, researchers usually need data to test and verify their hypotheses or models. We found that authors of the reviewed papers have used one of the two data types: real data and hypothetical data. We grouped data gathered through field studies, such as questionnaires and observations, studies of archival records, or studies of an actual emergency under *real data*. Hypothetical data is when researchers use some reasonable numbers due to many reasons, such as lack of archival data.

### Methodology

In the reviewed papers, researchers have used one or several qualitative or quantitative methods to investigate their intended problem. Within studies using quantitative methods, we found the following methods: cost-effectiveness analysis, mathematical programming, simulation, and statistical analysis. We found the following qualitative methods in the reviewed studies: focus group; interview; meta-analysis; pretest-posttest study (e.g., pretest-posttest cross-sectional design); prospective studies; real-life experiments including randomized trial, non-randomized trial, in-practice simulation, and training courses; retrospective studies; survey; and thematic analysis. For those works with more than one method (e.g., a retrospective study has been followed by a statistical analysis), we have categorized the work based on the method we considered the main one.

## Pre-EMS services

Tasked with providing timely pre-hospital medical care and saving lives of patients outside of hospitals, EMS are important actors in the healthcare system (Pozner et al. 2004; Ingolfsson 2013). In the majority of cases, ambulances with paramedics, emergency medical technicians or assistants, or registered nurses are dispatched to patients. After an initial treatment, if necessary, they can potentially take patients to hospitals and medical centers. In order to fulfil response time thresholds with a reasonable number of resources, ambulances must be placed efficiently. In EMS logistics, locating ambulances and ambulance bases is probably the most important planning problem, at least the one that has been studied the most. While in general the aim is to reach patients as fast as possible in case of an emergency, different objectives are used in ambulance location problems, such as maximizing coverage, minimizing response times, and maximizing survival probabilities. Further information about EMS logistics and planning problems can be found in Aringhieri et al. (2017), Reuter-Oppermann et al. (2017) and Bélanger et al. (2019), for example.

Already for some time, EMS providers worldwide have been facing two major challenges that lead to resource shortage problems: (1) budget cutbacks and (2) centralization of resources leading to longer response times to sparsely populated areas (Matinrad 2019). Additionally, in some countries fewer people are willing to work as professionals in EMS, and therefore, a shortage of human resources exists as well (DRK-Landesverband Baden-Württemberg e.V. and DRK-Landesverband Badisches Rotes Kreuz e.V 2018; Uppal and Gondi 2019). Thus, pre-EMS services have been introduced in the pre-hospital healthcare system and are utilized more frequently. In general, pre-EMS services, including human resources and equipment, have been facing rising attention during recent years especially since 2010 (see Fig. 3). These services have not traditionally been part of emergency response systems and nowadays are used prior to the arrival of an (EMS) ambulance.

**Fig. 1:**
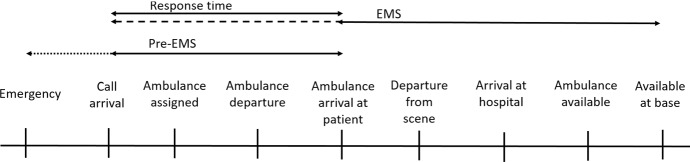
Pre-EMS services in the emergency response process

The classical emergency response process starts with the call arrival (e.g., Reuter-Oppermann et al. (2017); Aringhieri et al. (2017)). As shown in Fig. 1, pre-EMS services might be provided at the scene of an emergency during the EMS *response time*, until an ambulance arrives. In case of bystanders at the scene, pre-EMS services might even start before the call takes place. If resources like volunteers or drones are assigned by the coordination center, it is only valuable if they arrive (significantly) before the ambulance.

**Fig. 2:**
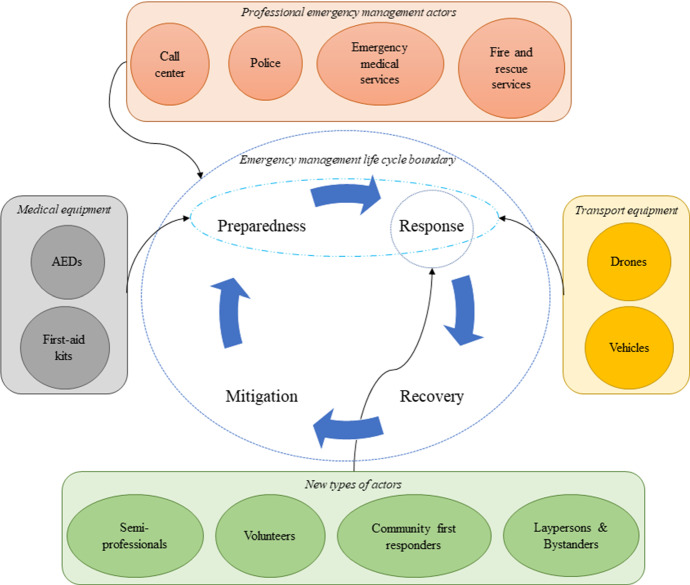
Actors and equipment involved in emergency management life cycle

In Fig. 2 we show human resources (i.e., actors) and equipment contributing to emergency management. The center of this figure displays the emergency management life cycle, which is seen widely in disaster management literature (e.g., Coppola (2006); Nikbakhsh and Farahani (2011)). This cycle is, however, applicable to the management of daily emergencies as well (Matinrad 2019). As this figure shows, the emergency management life cycle consists of four phases of mitigation, preparedness, response, and recovery. While professional emergency management actors (i.e., ambulances, call centers, fire and rescue services (FRS), and police) are involved in all four phases to a greater or lesser extend, new types of actors (i.e., community first responders, laypersons and bystanders, semi-professionals, and volunteers) are mostly involved in the response phase. These human resources need some equipment in their response operations. The equipment used for medical emergencies, classified into two categories of medical equipment (i.e., first-aid kits and AEDs) and transport equipment (i.e., drones and vehicles), are also presented in this figure. These equipment are involved in both phases of preparedness and response. It should be noted that medical equipment in this figure are only those used by the new types of actors. The arrows coming from outside of *emergency management life cycle boundary* connects each of the resource categories to the phase(s) they are involved in.

**Fig. 3:**
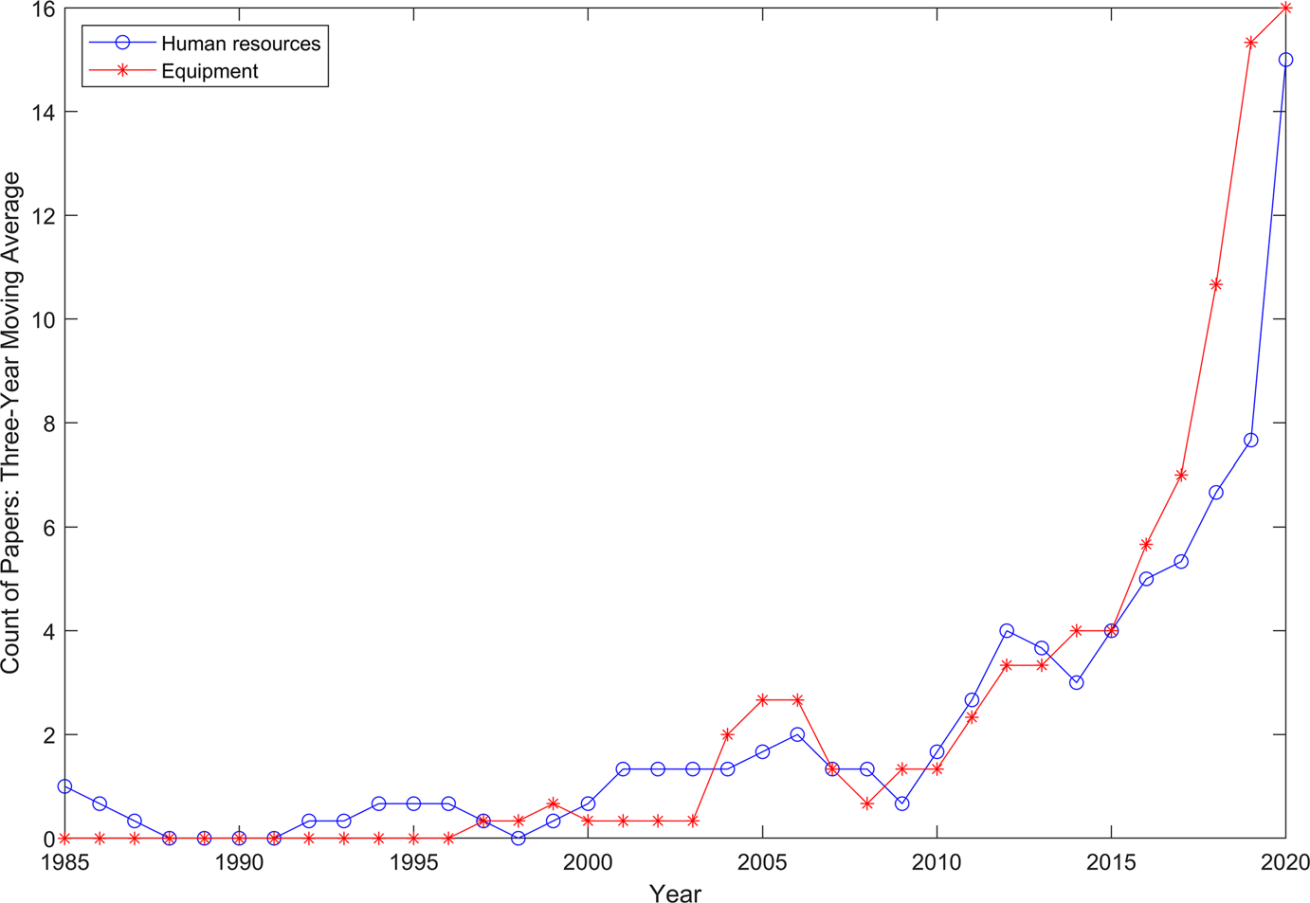
Three-year moving average of number of papers published in all journals and conference proceedings

Based on the resource type that researchers have focused on, we grouped studies on pre-EMS services into two main categories of *human resources* and *equipment*. We used the three-year moving average for both categories to inspect the rate of growth of pre-EMS services literature and present the results in Fig. 3. As can be seen in this figure, both human resources and equipment have incremental trends with a continuously positive rate of growth since 2014. These trends clearly show a growing interest in pre-EMS services especially in the recent years.

In the remainder of this section, we will provide an overview of studies on human resources and equipment that are used in pre-EMS services in Sects. 3.1 and 3.2, respectively. Then, in Sect. 3.3 we will further analyze publications that have used mathematical programming as their main methodology.

### Human resources

We categorize human resources that are used in pre-EMS services into four main groups:

- Community first responders (CFR)
- First responders
- Laypersons and bystanders
- Volunteers.

The subcategory *CFR* includes people who are not part of the professional emergency management system, but know how to handle (some, if not all) medical emergencies, because they either have received basic medical training (e.g., school nurses) or they are medically educated such as nurses and doctors. In some literature and initiatives this category might be also known as *semi-professionals* (e.g., Granberg et al. 2016, 2017; Yousefi Mojir and Pilemalm 2013). *First responders* include FRS and police. These organizations are already active in professional emergency management systems but are included in this list, because their primary roles are non-medical. In this context, FRS and police respond to some medical emergencies and provide help to patients prior to the arrival of EMS. According to the Merriam-Webster dictionary, *laypersons* are those people who “do not belong to a specif profession or are not expert in some fields”. Therefore, in the context of pre-EMS services, laypersons are neither affiliated with EMS nor have any formal medical training. According to Nord (2017), *bystanders* can include both laypersons or medically educated people (e.g., off-duty healthcare personnel). Volunteers are people who *volunteer* to help in case of emergencies and depending on their integration within the emergency response systems, they might be well trained, equipped, and experienced (e.g., firefighter volunteers) or just have some minimum level of training (Matinrad 2019). In the literature, we found that some researchers use these terms interchangeably. For instance, they have used laypersons while they are actually referring to volunteers. Therefore, we categorized the 127 human resources papers here based on the actual subcategory that researchers have referred to rather than solely relying on the term they have used in their studies.

In the remaining part of this section, we first present general attributes of human resources within pre-EMS services in Sect. 3.1.1. Then, in Sect. 3.1.2 we provide cross tabulations of four main categories of these resources outlined in Sects. 2 and 3.1.

#### General attributes

In Table 1 we list the number of papers per year for each subcategory of human resources. As it can be seen from this table, most papers in human resources are published after 2000 with the highest number published in 2020. Laypersons and bystanders have the highest number of publications (44), followed by first responders (40) and volunteers (37). CFR with six papers has the lowest number of publications within human resources. The reason for the few publications focusing on CFR might be because this group can also be considered under the subcategory *volunteers*.

**Table 1:** Number of papers by subcategory per year for human resources

Subcategory	1982–2000	2001	2002	2003	2004	2005	2006	2007	2008	2009	2010	2011	2012	2013	2014	2015	2016	2017	2018	2019	2020	Total
CFR	1	0	0	0	0	0	0	0	0	0	0	0	0	1	0	1	1	1	0	1	0	6
First responders	4	1	1	2	1	1	0	0	0	2	3	0	1	2	2	3	0	6	0	8	3	40
Laypersons and Bystanders	3	0	0	0	0	1	0	0	0	1	1	1	0	0	1	0	1	2	6	10	17	44
Volunteers	2	0	0	0	1	0	0	2	0	0	1	3	1	0	4	1	2	2	2	7	9	37
Total	10	1	1	2	2	2	0	2	0	3	5	4	2	3	7	5	4	11	8	26	29	127

Within each subcategory, researchers have focused on a group of actors that are relevant to that subcategory and we call them “focus group” in this study. In Table 2 we present the number of papers relevant to each subcategory and focus group. In this table, medical staff includes general practitioners, general surgery residents, surgical trainees, medical students and research trainees, and off-duty EMS personnel. The focus group *multiple actors* consists of FRS and police officers that can be accompanied by one or several other actors including private security personnel, volunteers, and bystanders. *Volunteers* as well as *laypersons and bystanders* have *civilians* as their main focus group. The classification of the reviewed works in this table is dependent on the aim and structure of the studies, besides the relation between the subcategory and the focus group. Some additional observations regarding these classifications include the following.

- Even though medical staff is generally a focus group related to CFR, sometimes it relates to a different subcategory based on the aim of the study. In one study surgical trainees were used to simulate a tourniquet application in order to examine tourniquet instructions that will be used by laypersons and bystanders. In another study off-duty EMS personnel have acted as volunteers to investigate a volunteer response program. While the subcategory of the former study is *laypersons and bystanders* and that of the latter is *volunteers*, both studies have *medical staff* as their focus group.
- FRS is a focus group generally associated with the subcategory *first responders*. However, in a study researchers have investigated the development of a mobile-volunteer program and used off-duty FRS as volunteers in the trial. Consequently, even though the focus group of this study is categorized under *FRS*, because the aim of the investigation was related to volunteers, its subcategory is *volunteers*.
- Usually the subcategory *CFR* is associated with one of focus groups that are not affiliated with EMS but have some level of medical training (e.g., school nurses). However, in one study, in order to investigate the possibility of introduction and implementation of a CFR system, researchers have sent out a survey to 1350 residents. Therefore, as the aim of the study concerns CFR, the study is categorized as *CFR*, but the focus group is *civilians*.

**Table 2:** Number of papers by subcategory and focus group

Subcategory versus Focus group	Casino security officers	Civilians	FRS	Home-care providers	Medical staff	(Motorcycle) Taxi riders	Multiple actors	Police officers	School nurses	Total
CFR	1	1	0	1	2	0	0	0	1	6
First responders	0	0	18	0	0	0	15	7	0	40
Laypersons and Bystanders	0	38	0	0	1	3	1	0	1	44
Volunteers	0	35	1	0	1	0	0	0	0	37
Total	1	74	19	1	4	3	16	7	2	127

In Table 3 we provide a closer look at the number of published papers based on each type of emergency in each of the continents. As can be seen from this table, 51 papers (40%) are conducted within Europe. From these publications, 15 studies are related to Sweden, six to the Netherlands and six to the UK. European researchers have focused mostly on OHCA, while those in North America have studied bleeding and OHCA almost equally. The studies on bleeding in North America are a result of the “Stop the Bleed” campaign that was initiated after a number of mass casualty incidents in the USA. North America has the second highest number of studies, 45 papers (35%), with 38 studies related to the USA. 16 papers (13%) are related to Asia and Oceania followed by eight papers (six percent) from Africa, in which trauma is the most studied type of emergency. A lack of sufficient professional resources combined with road traffic injuries among major types of daily emergencies can be the reason for this focus in Africa. In seven papers (six percent) researchers have not stated on which country or region they have focused. We found no study focusing on South America. Even though some of the *unknown* studies could be related to South America, no country from this continent is explicitly mentioned in any of the reviewed works.

**Table 3:** Number of papers by continent and emergency type

Continent versus Emergency type	Bleeding	General	OHCA	Trauma	Total
Africa	0	1	0	7	8
Asia and Oceania	1	0	14	1	16
Europe	0	11	39	1	51
North America	19	5	17	4	45
Unknown	0	0	5	2	7
Total	20	17	75	15	127

**Fig. 4:**
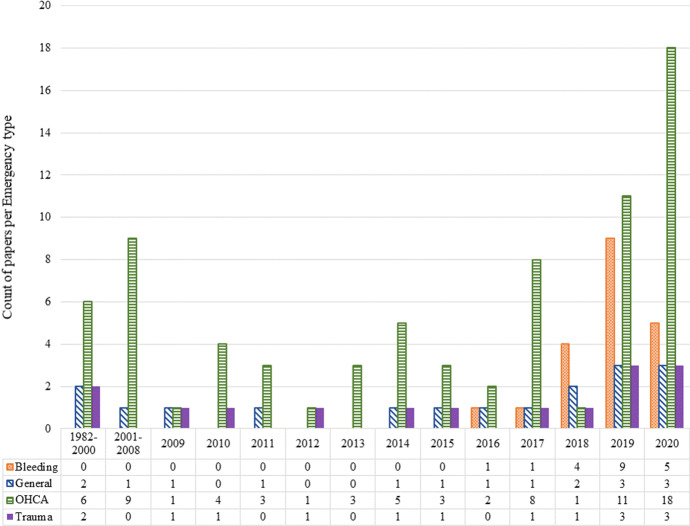
Number of papers per year by emergency type

To see how the focus on different types of emergencies has changed over the years, we present Fig. 4. As shown in this figure, the number of studies related to trauma is almost the same across all years with a slight increase in 2019 and 2020. Bleeding has faced a rising interest since 2016 with the highest number of publications related to 2019. OHCA, on the other hand, has been having a different trend with fewer publications in the middle years (i.e., from 2009 to 2016) and the most publications in 2020.

#### Cross tabulation

With regard to the four main categories detailed in Sects. 2 and 3.1, in this section we present five cross tabulations: (1) emergency type versus subcategory, (2) subcategory versus data type, (3) data type versus methodology, (4) subcategory versus methodology, and (5) emergency type versus methodology. With the help of these tables we draw some insights regarding the literature on human resources.

**Fig. 5:**
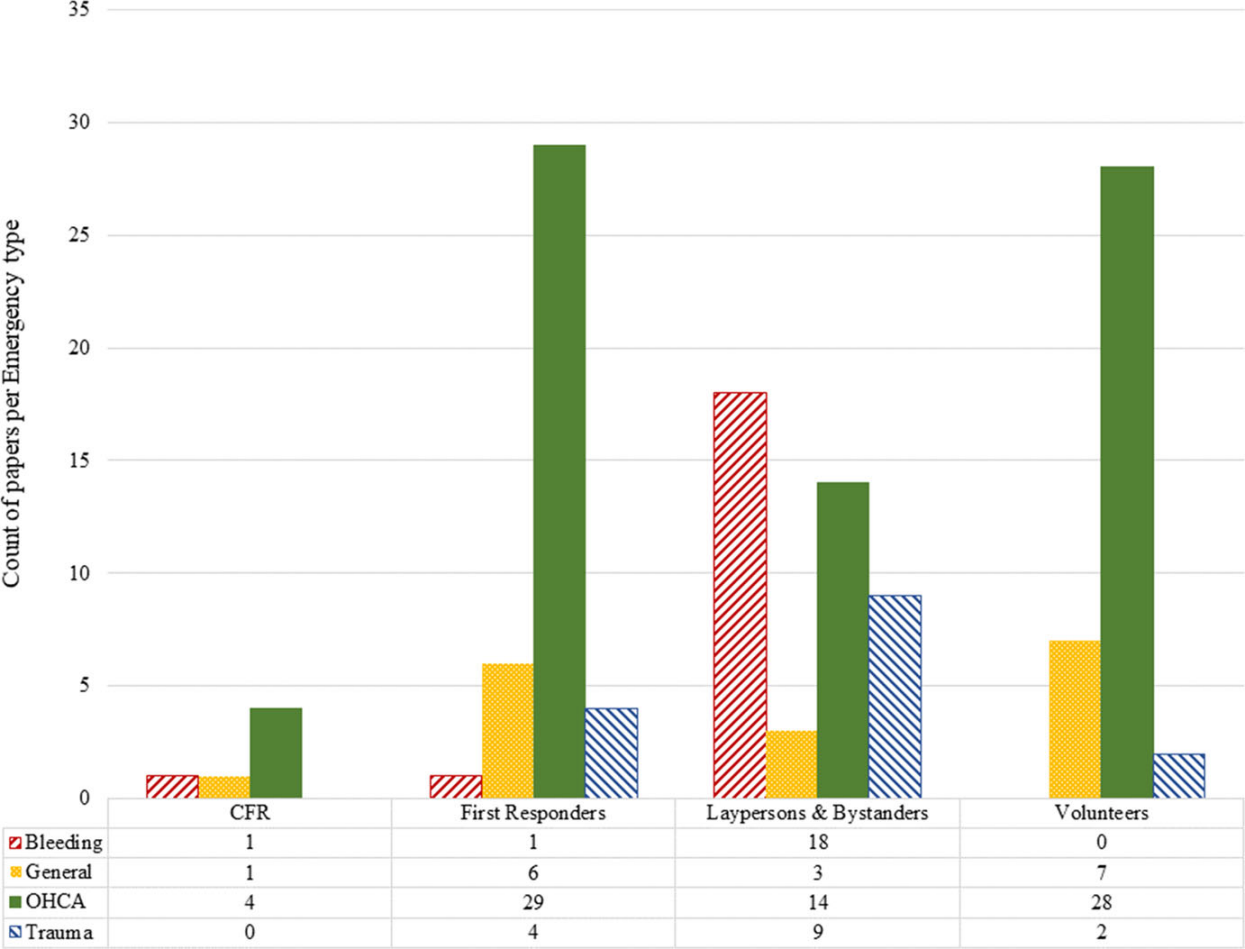
Number of papers by emergency type per subcategory

In Fig. 5 we demonstrate both the cross tabulation of emergency type versus subcategory (as a numeric table) and the distribution of published papers in each subcategory by emergency type (as a diagram). As we can see from this figure, the focus of most papers is on OHCA, 29 papers considering the use of first responders to respond to this type of emergency, followed by volunteers (28 papers). Laypersons and bystanders are considered in nine papers in cases of trauma, but have been studied twice in case of bleeding (18 papers). These resources are considered in 14 papers with the focus on OHCA. CFR have been studied mostly in case of OHCA (four papers). Papers in the *general* category, for which authors of related papers have not clearly mentioned the type of emergency, have almost equally focused on volunteers (seven papers) and first responders (six papers), and have studied the use of laypersons and bystanders in three papers and CFR only in one paper.

Based on these observations it seems that the use of first responders as well as volunteers in response to emergencies involving bleeding, when people usually apply tourniquet (or similar tools such as a belt), is an area to focus on. In cases in which shooting and knife attacks occur, first reponders, especially the police, are often the first at the scene of the event. Thus, if they already know how to stop a bleeding properly, it can help save lives of the patients. It is understandable that no study has focused on the use and dispatch of volunteers to manage bleeding. If the bleeding has been the result of a shooting, for example, the presence of volunteers at the scene can not only crowd the scene, but can also endanger their lives, especially if the situation is still on-going at the time of their arrival. Nevertheless, if the bleeding is a consequence of a common daily emergency (e.g., a bike accident), volunteers with the adequate skills for handling bleeding can be helpful. Overall, few studies have considered trauma as their emergency type. The use of different resource types for this emergency type is reasonable, for example in road traffic emergencies, in which trauma patients can be present. In such cases, first responders can be sent to the scene of the event if they will arrive sooner than the EMS to provide medical care. However, future work is needed to study if these resources (volunteers and first responders for bleeding cases and first responders in trauma cases) can contribute to saving lives, analogously to how it has been studied for OHCA.

We list all references based on subcategory and emergency type in Table 8. This table can be helpful for finding relevant references in the cross section of subcategories and emergency types. In this table each column is allocated to one type of emergency and each row represents a subcategory. In each cell we include papers published in different years that focus on a specific combination of subcategory and emergency type rather than the total number of publications. The last row and column give the total number of published papers per emergency type and subcategory, respectively.

Table 4 shows that the majority of papers (115) have used some type of real data. Researchers have gathered this data directly, for example through field research (i.e., primary data sets), or they have obtained it through archival data sets (i.e., secondary or tertiary data sets). In two papers researchers have used hypothetical data, while in 10 papers researchers have used no data.

**Table 4:** Number of papers by subcategory and data type

Subcategory versus Data type	Hypothetical	Real	No data	Total
CFR	0	6	0	6
First responders	0	38	2	40
Laypersons and Bystanders	1	39	4	44
Volunteers	1	32	4	37
Total	2	115	10	127

In Table 5 we present the cross tabulation of data type versus primary methodology. In this table we have classified papers based on the method we found as the primary methodology used in that work. In some papers multiple methods have been used to obtain and analyze data. For instance, researchers have conducted a survey and then used simple statistical analysis techniques to quantify and further analyze the data. In addition, Table 5 includes both data gathering and data analysis techniques. Comparing the results in Tables 4 and 5 we can see that the 10 papers, in which no data has been used, are all related to review papers, of which one has focused on reviewing an app-based system (i.e., the GoodSAM app in the UK). By checking Table 5 we can also see that one of the papers using hypothetical data applies cost-effectiveness analysis and the other one mathematical programming as their methods. Some qualitative methods such as focus group, survey, and interview result in real data. However, the results of our review, as presented in Table 5, show that researchers have also (mostly) used real data for quantitative methods such as mathematical programming and simulation. Some additional observations include the following.

**Table 5:** Number of papers by data type and primary methodology

Data type versus Methodology	Cost-effectiveness analysis	Focus group	Interview	Mathematical programming	Meta-analysis	Pretest-posttest study	Prospective studies	Real-life experiment	Retrospective studies	Review	Simulation	Statistical analysis	Survey	Total
Hypothetical	1	0	0	1	0	0	0	0	0	0	0	0	0	2
Real	2	2	5	1	1	1	24	34	19	0	1	17	8	115
No data	0	0	0	0	0	0	0	0	0	10	0	0	0	10
Total	3	2	5	2	1	1	24	34	19	10	1	17	8	127

- The number of papers that have used one of the quantitative methods as their primary method accounts for 18% of all studies (i.e., 23 out of 127 papers). The high number of qualitative studies may be because within the medical research area conducting a trial or real-life simulation, or holding focus groups and interviews, in which people are involved in the study, are more common methods. In the healthcare area, even when a mathematical model is proposed and tested on real data, it should still be tested in a randomized trial in real life to ensure the analytically determined results are sound and reliable in practice as well. However, if prior to such a qualitative method (e.g., real-life experiment) the assumptions are modeled and tested quantitatively, it can potentially help reduce associated costs and increase the probability of success. In addition, using real data contributes to building a model or theory that can reflect the reality better and their results can be of higher trust for emergency managers and administrators.
- The methodology *survey* entails the entire process of data gathering (e.g., questionnaire, interview) and analyzing that data, while *questionnaire* is only a data gathering method. However, we found in the reviewed papers that researchers sometimes use survey and questionnaire interchangeably. Therefore, we have categorized papers using either of them under the same method category, *survey*.

In Table 6 we present the cross tabulation of subcategory versus methodology. In this table we present all methods used in each reviewed paper. For example, in one paper researchers have conducted a randomized control field experiment (listed under Real-life experiment) on volunteers and in case of trauma. Then these authors have used a survey and interviews to complement their research data, and eventually utilized statistical analysis to further analyze and draw their results. Therefore, methods used in this study are counted in all categories of *real-life experiment*, *survey*, *interview*, and *statistical analysis*, while we considered real-life experiment as the primary methodology of this work. It is noticeable that while only 17 papers used statistical analysis as their primary methodology (see Table 5), the number of papers that use this method in their work as a primary or complementary method sums up to 82. Our findings indicate that the majority of studies on laypersons and bystanders use real-life experiment (20 papers, of which 16 papers used it as their primary methodology), disregarding the use of statistical analysis as a complementary method. In the same way, researchers have studied volunteers by using mainly real-life experiments (10 papers). The studies on first responders have mostly relied on prospective studies and retrospective studies, eight papers each.

**Table 6:** Number of papers by subcategory and all applied methodologies

Subcategory versus Methodology	Cost-effectiveness analysis	Focus group	Interview	Mathematical programming	Meta-analysis	Pretest-posttest study	Prospective studies	Real-life experiment	Retrospective studies	Review	Simulation	Statistical analysis	Survey	Thematic analysis	Total
CFR	0	0	0	0	0	1	2	1	1	0	0	4	1	0	10
	2	3	2	0	1	1	8	6	8	2	0	26	4	0	63
	1	3	3	0	0	2	9	20	4	4	0	28	7	1	82
Volunteers	0	0	5	2	1	0	5	10	6	4	1	24	5	1	64
Total	3	6	10	2	2	4	24	37	19	10	1	82	17	2	219

Table 7 is a cross tabulation of emergency type versus methodology. In this table we present all methodologies used in each paper, similar to Table 6. If we disregard statistical analysis, which has served greatly as a complementary method (65 papers out of 82 papers), in case of bleeding, 12 studies have applied real-life experiment and no quantitative method has been used to study this emergency type. OHCA has been studied mostly using prospective studies and retrospective studies, 14 papers each and all as the primary method, and real-life experiment (13 papers, of which 10 papers used it as their primary methodology). However, researchers not only have applied other types of qualitative methods (e.g., survey (nine papers)) to investigate issues related to OHCA, but they have also used quantitative methods (e.g., mathematical programming (two paper) and simulation (one paper)). Trauma is studied qualitatively with statistical analysis serving as a complementary method, except in one paper, in which it has been used as a primary method.

**Table 7:** Number of papers by emergency type and all applied methodologies

Emergency type versus Methodology	Cost-effectiveness analysis	Focus group	Interview	Mathematical programming	Meta-analysis	Pretest-posttest study	Prospective studies	Real-life experiment	Retrospective studies	Review	Simulation	Statistical analysis	Survey	Thematic analysis	Total
Bleeding	0	1	0	0	0	1	6	12	0	0	0	16	3	1	40
General	0	1	2	0	1	0	3	3	5	2	0	8	1	0	26
OHCA	3	1	3	2	1	0	14	13	14	6	1	51	9	1	119
Trauma	0	3	5	0	0	3	1	9	0	2	0	7	4	0	34
Total	3	6	10	2	2	4	24	37	19	10	1	82	17	2	219

**Table 8:** References (list of authors) for subcategories by emergency types

Subcategory versus Emergency type	Bleeding	General	OHCA	Trauma	Total
CFR	Latuska et al. (2019)	Campbell and Ellington (2016)	Valenzuela et al. (2000), Toyokuni et al. (2013), Hansen et al. (2015b) and Barry et al. (2017)		6
First responders	Ali et al. (2019)	Berringer et al. (1999), Quinn et al. (2009), Boland et al. (2015), Lichtenhahn et al. (2019), Tamminen et al. (2019) and Svensson et al. (2020)	Shuster and Keller (1993), White et al. (1998), Jermyn (2000), Smith et al. (2001), Myerburg et al. (2002), Lerner et al. (2003b), Lerner et al. (2003a), Kooij et al. (2004), De Vries et al. (2005), Høyer and Christensen (2009), Boyle et al. (2010), Craig et al. (2010), Hess and White (2010), Sund et al. (2012), Husain and Eisenberg (2013), Saner et al. (2013), Nordberg et al. (2014), Winship et al. (2014), Hansen et al. (2015a), Boland et al. (2017), Caputo et al. (2017), Claesson et al. (2017b), Stein et al. (2017), Hasselqvist-Ax et al. (2017), Hasselqvist-Ax et al. (2019), Nehme et al. (2019), Oving et al. (2019), Raun et al. (2019), Krammel et al. (2020)	Nilsson et al. (2015), Chokotho et al. (2017), Lukumay et al. (2019) and Delaney et al. (2020)	40
Laypersons and Bystanders	Goolsby et al. (2016), Forsyth et al. (2017), AlSabah et al. (2018), Dhillon et al. (2019), Goolsby et al. (2018), Goralnick et al. (2018), Ross et al. (2018), Goolsby et al. (2019), Lei et al. (2019), Lowndes et al. (2019), McCarty et al. (2019a), McCarty et al. (2019b), Zwislewski et al. (2019), Andrade et al. (2020), Nanassy et al. (2020), Portela et al. (2020), Scott et al. (2020) and Stadeli et al. (2020)	Geduld and Wallis (2011), Curran et al. (2018) and Lamote et al. (2020)	Cummins et al. (1991), Groeneveld and Owens (2005), Herlitz et al. (1994), Karam et al. (2017), Brady et al. (2019), Blewer et al. (2020), Haskins et al. (2020), Kappus and McCullough (2020), Kim et al. (2020), Riva et al. (2020), Tay et al. (2020), Chen et al. (2020), Hatakeyama et al. (2020) and Shimamoto et al. (2020)	Brodsky (1984), Jayaraman et al. (2009), Murad and Husum (2010), Callese et al. (2014), Delaney et al. (2018), Heidari et al. (2019), Tatebe et al. (2019), Eisner et al. (2020) and Hancock et al. (2020)	44
Volunteers		Kay and Myrick (1982), Naths et al. (2007), Roberts et al. (2014), Phung et al. (2017), Phung et al. (2018), Ramsell et al. (2019), Schwartz et al. (2020)	Culley et al. (2004), Groh et al. (2007), Rørtveit and Meland (2010), Ringh et al. (2011), Scholten et al. (2011), Narikawa et al. (2014), Yonekawa et al. (2014), Zijlstra et al. (2014), Ringh et al. (2015), Cairns et al. (2016), Capucci et al. (2016), Pijls et al. (2016), Smith et al. (2017c), Berglund et al. (2018), Auricchio et al. (2019), Barry et al. (2019), Del Pozo et al. (2019), Gross et al. (2019), Matinrad et al. (2019), Pijls et al. (2019), Andelius et al. (2020), Blackwood et al. (2020), Derkenne et al. (2020), Jonsson et al. (2020), Rao et al. (2020) Sarkisian et al. (2020), Stroop et al. (2020), Scquizzato et al. (2020)	Myrick et al. (1983) and Sun and Wallis (2012)	37
Total	20	17	75	15	127

### Equipment

Besides human resources different types of equipment are utilized in pre-EMS services as well. Two main equipment types used in these services are AEDs and drones, also known as unmanned aerial vehicles (UAVs). We found 90 relevant papers on AEDs, 22 on drones, and 19 publications that simultaneously targeted drones and AEDs. In this section, we provide an overview of all these papers, studying equipment qualitatively or quantitatively. The papers using mathematical programming among quantitative works will be detailed in Sect. 3.3.

We can categorize AED-related papers under two main topics: (1) impact and usefulness of AEDs, and (2) location of AEDs. Researchers whose work categorizes under the first topic mostly have investigated the survival of OHCA patients. They have considered measures such as survival until discharge, and 30-day survival for patients for whom AEDs have been used. Researchers who have focused on the location of AEDs have investigated problems such as whether AEDs should be placed in public areas or not, whether AEDs should be located inside or outside of (high) buildings, or the impact of AED distributions and their access in urban versus rural areas. AEDs’ accessibility and barriers such as awareness or willingness to use an AED have also been considered in the literature. Equipping basic life support ambulances with AEDs and transport of AEDs using public transportation are other investigated areas concerning AEDs.

Drones in pre-EMS services are used either to transport necessary equipment, such as AEDs and medicine, or to prevent people from drowning by helping them afloat. Another use of drones is to scan or photograph a scene or area in order to help with locating a drowning victim. Having drones transport AEDs to an emergency scene has been investigated as an addition or an alternative strategy to locate stationary AEDs.

In Table 9 we provide an overview of the number of papers published per year for equipment. While studies targeting the use of AEDs have already been published as early as the 1980s, relevant publications on drones within pre-EMS services could only be found from 2015. The highest number of papers related to drones was published in 2020.

**Table 9:** Number of papers by equipment subcategory per year

Subcategory	1982–2000	2001	2002	2003	2004	2005	2006	2007	2008	2009	2010	2011	2012	2013	2014	2015	2016	2017	2018	2019	2020	Total
AEDs	2	1	0	5	3	0	1	1	2	1	4	5	1	6	5	5	8	7	11	6	16	90
Drones	0	0	0	0	0	0	0	0	0	0	0	0	0	0	0	0	0	5	6	4	6	22
AEDs and Drones	0	0	0	0	0	0	0	0	0	0	0	0	0	0	0	1	2	4	3	2	8	19
Total	2	1	0	5	3	0	1	1	2	1	4	5	1	6	5	6	10	16	20	12	30	131

We show the number of publications for the equipment types per continent in Table 10. The majority of publications focusing on North America and Europe correspond to 63% (83 papers) of the studies. In North America, 28 papers focus on the USA, of which five papers have studied *AEDs and drones* and three papers *drones*. Within the European countries the highest numbers of publications target Denmark (eight papers), the Netherlands (seven papers), and Sweden (seven papers), with Denmark and the Netherlands focusing solely on the use of AEDs and Sweden on all three categories of equipment. All papers but one related to *Asia and Oceania* have focused on AEDs with the highest number of publications related to Japan and Taiwan (seven and five papers, respectively). We found only three publication for Africa (i.e., United Republic of Tanzania and Republic of Guinea). Similar to human resources, we found no paper for South America (see Sect. 3.1). In addition, 22 papers either did not state a country or targeted many countries worldwide (e.g., in a review paper) and are thus listed as “unknown” in the table.

**Table 10:** Number of papers by continent and equipment subcategory

Continent versus Equipment subcategory	AEDs	Drones	AEDs and Drones	Total
Africa	0	3	0	3
Asia and Oceania	22	1	0	23
Europe	29	7	5	41
North America	29	5	8	42
Unknown	10	6	6	22
Total	90	22	19	131

Related to the type of emergencies, the AED-related papers (i.e., *AEDs and drones*, and *ADEs*) all targeted OHCA. Publications on drones, however, considered several emergency types including drowning (four papers), trauma (one paper), elderly patients falling (one paper), epilepsy (one paper), and OHCA (one paper). 14 papers addressed the use of drones in general.

In Table 11 we reference all publications on equipment with rows showing types of equipment and columns displaying the main methods used by researchers for these studies (i.e., qualitative, quantitative, review). As we focus on studies using mathematical programming in Sect. 3.3, we divide quantitative studies into *Mathematical Programming* and *Other* to make it easier to find references using mathematical programming. As shown in this table, 83 papers have used one type of quantitative method, while 31 paper have used a qualitative type of method. In addition, in 17 papers researchers have reviewed existing literature on different types of equipment.

**Table 11:** References (list of authors) for main methods by equipment subcategories

Equipment subcategory versus Main method	Qualitative	Quantitative		Review	Total
		Mathematical programming	Other		
AEDs	Jorgenson et al. (2003), Andre et al. (2004), Harve and Silfvast (2004), Bahr et al. (2010), Sakai et al. (2011), Schober et al. (2011), Toresdahl et al. (2013), Deakin et al. (2014), White et al. (2016), Elrod et al. (2017), Holmberg et al. (2017), Smith et al. (2017a), Kua et al. (2018), Briard et al. (2019), Morgan et al. (2019), Fortington et al. (2020), PARK and UHM (2020), Qutub (2019), Schmidt-Polończyk and Jaskula (2020)	Mandell and Becker (1996), Myers and Mohite (2009), Dao et al. (2012), Tsai et al. (2012), Chan et al. (2013), Siddiq et al. (2013), Huang and Wen (2014), Bonnet et al. (2015), Chan et al. (2016), Dahan et al. (2016), Lin et al. (2016), Sun et al. (2016), Chan (2017), Chan et al. (2018), Sun et al. (2018), Tierney et al. (2018), Lee et al. (2019), Rao et al. (2019), Derevitskii et al. (2020), Hajari et al. (2020) and Lorenzo et al. (2020), Yang et al. (2020)	Nichol et al. (1998), Ross et al. (2001a), Cram et al. (2003), Rauner and Bajmoczy (2003), Roccia et al. (2003), van Alem et al. (2003), Portner et al. (2004), Sharieff and Kaulback (2007), Moore et al. (2008), Folke et al. (2010), Kitamura et al. (2010), Rea et al. (2010), Berdowski et al. (2011) Rea et al. (2011), Hansen et al. (2013), Levy et al. (2013), Nielsen et al. (2013), Ohta et al. (2014), Murakami et al. (2014), Agerskov et al. (2015), Moon et al. (2015), Moran et al. (2015), Nelson et al. (2015), Henriksen et al. (2016), Kiyohara et al. (2016), Karam et al. (2017), Sun et al. (2017), Zijlstra et al. (2017), El-Assaad et al. (2018), Fredman et al. (2018), Nas et al. (2018), Sondergaard et al. (2018), Zijlstra et al. (2018), Karlsson et al. (2019), Wang et al. (2019), Chua et al. (2020), Griffis et al. (2020), Grunau et al. (2020), Kobayashi et al. (2020), Fan et al. (2020), Moriwaki et al. (2020), Stieglis et al. (2020), Xu et al. (2020)	Maisch et al. (2006), Mell and Sayre (2008), Ströhle et al. (2014), Samani and Zhu (2016), Smith et al. (2017b) and Ringh et al. (2018)	90
Drones	Claesson et al. (2017c), Bäckman et al. (2018), Clark et al. (2018), Krey (2018), Seguin et al. (2018), Khan and Neustaedter (2019), Gupta et al. (2020),	Dorling et al. (2017), Scott and Scott (2017), Wang et al. (2017), Walia et al. (2018), Mao et al. (2019), Kartawijaya et al. (2019), Anastasiou et al. (2020), Mateen et al. (2020), Maria Elena Nenni (2020)	Claesson et al. (2020), Fakhrulddin and Gharghan (2020)	Balasingam (2017), Otto et al. (2018), Konert et al. (2019) and Scott and Scott (2020)	22
AEDs and Drones	Claesson et al. (2017a), Sanfridsson et al. (2019), Cheskes et al. (2020), Sedig et al. (2020), Zègre-Hemsey et al. (2020)	Claesson et al. (2016), Pulver et al. (2016), Boutilier et al. (2017), Pulver and Wei (2018), Bogle et al. (2019), Mackle et al. (2020), Wankmüller et al. (2020)		Thiels et al. (2015), Mark et al. (2017), Van de Voorde et al. (2017), Latimer et al. (2018), Zègre-Hemsey et al. (2018), Mermiri et al. (2020) and Shirane (2020)	19
Total	31	38	45	17	131

We detail the methods used for all the references in Table 12. As we can see in this table, mathematical programming (38 papers) and statistical analysis (39 papers) are mostly used as the primary methodology. While mathematical programming is used in studies related to all equipment types, researchers have used statistical analysis primarily only for studies on AEDs. In addition, reviews (17 papers) have a relatively high number. It is interesting to note that researchers have used real-life experiments relatively balanced studying different equipment types (i.e., AEDs (four papers), drones (three papers), and AEDs and drones (four papers)). However, despite the usefulness and flexibility of simulation approaches, researcher have rarely used computer simulation as the primary methodology (these are categorized under *mathematical programming* in Table 12) and very few have used it as a complementary method (see Table 13).

**Table 12:** Number of papers by equipment subcategory and primary methodology

Equipment subcategory versus Methodology	Cost-effectiveness analysis	Interview	Machine learning	Mathematical programming	Meta-analysis	Real-life experiment	Review	Statistical analysis	Survey	Total
AEDs	5	2	0	22	1	4	6	39	11	90
Drones	0	4	2	9	0	3	4	0	0	22
AEDs and Drones	0	1	0	7	0	4	7	0	0	19
Total	5	7	2	38	1	11	17	39	11	131

In terms of the data types used by researchers, we found that in all but three publications, excluding reviews, real data was used. The three papers, in which hypothetical data was used, researchers have applied mathematical programming as their primary methodology.

On the cross section of research on equipment and human resources in pre-EMS services, six papers related to drones transporting AEDs to an emergency explicitly mention the involvement of bystanders. With regard to literature on AEDs, different user groups including bystanders, volunteers, and first responders have been explicitly addressed. In one study researchers studied the use of AEDs by bystanders and discovered the importance of the AED user interface for the ability and success of a bystander to use an AED. In another study that reports on the use of AEDs in Europe, researchers found that out of the 36 studied European countries, in 11 of them only trained persons are allowed to use an AED. In addition, they stated that in 14 countries a few community responder programs exist. Also, one research group studied the location of AEDs for public use by bystanders and another one performed a survey with first responders from FRS concluding a lack of national standards and regulations for full integration of first responders programs into the EMS system.

### Quantitative studies in pre-EMS services

In this section we take a closer look at the studies using mathematical programming for both equipment and human resources. In Table 13 we present an overview of the 41 publications using mathematical programming as their primary methodology (i.e., three papers related to human resources and 38 to equipment). In this table rows include pre-EMS services and columns types of data, modeling characteristics and approaches (i.e., methods), objective functions, planning levels, and planning problems.

**Table 13:** Overview of papers using mathematical programming

Resource type	Data		Method							Objective				Planning level			Planning problem		
	Hypothetical	Real	ILP/MILP/MINLP	Heuristic/meta-heuristic	Deterministic	Stochastic/probabilistic	Robust	Multicriteria	Simulation	Min costs/resources	Max coverage	Max survival	Other	Strategic	Tactical	Operational	Location planning	Routing	Assignment
AEDs	3	21	15	7	16	2	1	5	2	3	16	2	5	22	0	1	22	0	1
Drones	3	3	5	3	4	1	0	0	2	2	0	1	3	6	0	3	6	3	0
AEDs and Drones	1	7	5	3	5	0	0	3	0	2	3	0	3	6	0	0	6	0	0
Human resources	1	2	2	0	2	0	0	0	1	1	0	1	2	1	0	2	0	0	3
Total	8	33	27	13	27	3	1	8	5	8	19	4	13	35	0	6	34	3	4

The majority of the models have used a deterministic approach for strategic location planning, dismissing the uncertainties inherited in emergency cases. Such uncertainties are related to, for instance, travel times, the availability of human resources, or the functionality of equipment. Therefore, a deterministic model could have less capability in depicting the reality, and thus, be less useful for practice. Stochastic/probabilistic or robust optimization that could include these uncertainties have hardly been applied. Only six papers have addressed the operational planning level and no paper has considered a planning problem on the tactical level.

Most of the models have used *coverage maximization* as the objective function. As most of these studies focused on the placement of AEDs, including coverage as the objective function is reasonable. However, considering that the pre-EMS services are used in response to medical emergencies, it can be beneficial to consider survival probability of patients in the modeling. Currently, only four out of 41 papers have considered survivability as their objective function. As shown by Erkut et al. (2008) for the ambulance location problem, the survival probability can be incorporated into the location problem as an objective function. 13 papers have used other objective functions, such as the time saved by using a drone or placing an AED compared to the arrival of the EMS or overall minimizing travel and response times.

The most common location model used by researchers is the maximal coverage location problem (MCLP), especially for the placement of AEDs. An alternative to this modeling could be the maximal survival location problem (MSLP) (Erkut et al. 2008). In this model, the focus is on the survivability of patients rather than coverage.

We found that simulation is used as a primary method only in three papers, one paper related to volunteers, one related to drones, and one to AEDs, and as a complementary method for two papers related to AEDs and drones (one for each). Mathematical models often need to make assumptions about reality in order to keep complexity and run times at a reasonable level that can still be handled. Simulations are thus a very important tool to analyze the computed solutions and account for the assumptions made in the models. They allow to vary input parameters and study the performance of the computed solutions (e.g., for increasing demand or time-dependent availability of volunteers).

While the majority of the researchers used real data, they usually focused on one city or region and did not test their approaches for different instances with varying characteristics.

Besides papers in which mathematical programming is used for modeling, we found statistical analysis as another dominating quantitative method used by researchers (see Tables 5 and 12). Researchers who have used statistical analysis have mostly applied one form of regression such as Poisson regression and logistic regression. They have (also) benefited from other statistical approaches such as descriptive statistics (e.g., mean, median, etc.), ROC analysis, Fisher test, $\chi ^2$ test, Mann-Whitney test, and *t* test.

## Pre-EMS services in different countries

In order to provide additional insights on pre-EMS services for EMS managers and decision makers as well as for researchers, we also want to briefly review pre-EMS initiatives in practice with a special focus on mobile phone applications (apps) that assign first responders or volunteers to emergencies, such as OHCA, and might also display location of available AEDs. Apps as part of pre-EMS initiatives in practice play an important role to instantiate the services and they also offer opportunities to easily integrate operations research approaches to efficiently design and manage the logistics. As we found in the papers reviewed in this work, researchers focusing on different countries (Australia, Austria, Belgium, Canada, Chad, Denmark, Finland, France, Germany, Guatemala, Iraq, Ireland, Italy, Japan, Kuwait, Madagascar, Malawi, the Netherlands, Norway, Republic of Guinea, Singapore, Sierra Leone, South Africa, Scotland, Spain, Sweden, Switzerland, Tanzania, Taiwan, Uganda, United Republic of Tanzania, the UK, and the USA) have started studying the effects of the use of pre-EMS services in medical emergency responses. Some of these countries have already started at least one form of these services in practice. In Table 14 we provide an overview of operational pre-EMS services in some of these countries, as documented through literature, as well as functional apps, where applicable. In a recent publication in the journal *Resuscitation*, Scquizzato et al. (2020) listed first responders apps and AED maps in Europe, while they did not provide any references or further information and also limited their consideration to countries within Europe. Prior to the work of Scquizzato et al. (2020), Oving et al. (2019) have performed a survey with 47 OHCA experts from 29 countries with the aim of providing an overview of first responder systems for OHCA in Europe. The authors state that the result of their investigations show a wide variation of initiatives, and they recommend that future research should more strongly focus on survival.

It should be noted that the majority of reported apps in Table 14 have been found by an extensive additional search.

**Table 14:** Overview of pre-EMS services in example countries

Country	Type of pre-EMS services			
	Equipment	Human resources	Existing App(s)	Reference(s)
Australia	AEDs	First responders	GoodSAM, St John First Aid Apps Australia, St John First Responder App Western Australia, Red Cross First Aid App	Morgan et al. (2019), Smith et al. (2001) and Winship et al. (2014); Good-SAM instant.help; Red Cross First Aid App Australia; St John First Aid Apps Australia; St John First Responder App Western Australia
Canada	AEDs, Drones	First responders	FirstAED Canada, Red Cross First Aid App Save a Life, FirstAED App, PulsePoint	Clark et al. (2018), Sharieff and Kaulback (2007) and Shuster and Keller (1993); Canadian Red Cross First Aid app; First AED App; First AED Canada; Pulse Point App; St John Save a Live AED Search and Register App
Denmark	AEDs	First responders	FirstAED App, TrygFonden Hjerteløber	Folke et al. (2010), Høyer and Christensen (2009) and Nielsen et al. (2013); First AED App; HeartRunner Sweden AB; TrygFonden Hjertestarter
Finland	AEDs	First responders, Volunteers	FirstAED App	Harve and Silfvast (2004) and Tamminen et al. (2019); First AED App; FirstAED
Germany	AEDs	First responders, Volunteers	Meine-Stadt-Rettet, Mobile Retter, FirstAED App, Defikataster, iHelp, Mobile Lebensretter, corhelper, Land-Rettung, KATRETTER, MV SCHOCKT	Gross et al. (2019), Lichtenhahn et al. (2019) and Naths et al. (2007) corhelper; Definetz e.V. Defikataster; First AED App; iHelp; KATRETTER; Land—Rettung; Meine Stadt Rettet; Mobile Retter; MobileLebensretter; MV SCHOCKT App
Italy	AEDs	Volunteers	Progetto Vita, DAE RespondER, DAEdove	Capucci et al. (2016); Associazione Progetto Vita; DAE RespondER; DAEdove
Netherlands	AEDs	First responders, Volunteers	Hartslagnu	Kooij et al. (2004), Slaa (2020), Schober et al. (2011) and Zijlstra et al. (2014); Hartslagnu
Singapore	AEDs	Volunteers	myResponder	Chua et al. (2020); myResponder (Accessed May 25, 2021)
Sweden	AEDs, Drones	CFR, First responders, Volunteers	Sms-livräddare	Bäckman et al. (2018), Claesson et al. (2016, 2017a), Granberg et al. (2016, 2017), Hasselqvist-Ax et al. (2017), Hollenberg et al. (2009), Nordberg et al. (2015, 2014), Ramsell et al. (2017), Ringh et al. (2011, 2015), Sanfridsson et al. (2019), Stenberg et al. (2014) and Yousefi Mojir and Pilemalm (2013) Akademiska Sjukhuset; Båstads Kommun; HeartRunner Sweden AB; Region Östergötland; Sms-livräddare
Switzerland	AEDs, Drones	First responders, Volunteers	Fondazione Ticino Cuore, CH Responder	Auricchio et al. (2019), Caputo et al. (2017), Krey (2018), Saner et al. (2013) and Stein et al. (2017); First Responder Kanton Bern
UK	AEDs	CFR, Volunteers	GoodSAM, iHelp	Deakin et al. (2014), Phung et al. (2017), Ross et al. (2001b) and Smith et al. (2017c, 2017a); GoodSAM instant. help; iHelp
USA	AEDs, Drones	First responders, Volunteers	GoodSAM, PulsePoint	Andre et al. (2004), Bogle et al. (2019), Boland et al. (2015), Culley et al. (2004), Elrod et al. (2017), Jorgenson et al. (2003), Mao et al. (2019), Moon et al. (2015), Rea et al. (2010), Roccia et al. (2003) and White et al. (2016); GoodSAM instant.help; Pulse Point App

As we can see from Table 14, *first responders* is the most frequent and common type of human resources that is used in practice in medical emergency responses in several countries. Other than first responders, *volunteers* are the second most employed human resource of pre-EMS services. Some of the apps, such as GoodSAM (GoodSAM instant.help 2020) and iHelp, are functional across several countries, while some others are used by one country or region, for example St John First Responder App Western Australia is used in Western Australia. In some of these apps, users in vicinity of an OHCA patient who can perform basic life support or use an AED are alerted and given the locations of the patient and AEDs in their area that they should pick up (e.g., Sms-livräddare 2020). These apps notify users and usually, based on their predefined algorithms, assign them one of the two tasks of (1) going directly to the patient or (2) picking up an AED en route to the patient. They are mostly designed based on the distance between the patient and each user as well as the distance of AEDs to users and the patient. Other apps only provide information for basic life support and AED locations for bystanders or first responders to use. Some of these apps, such as GoodSAM, are used by multiple human resources (e.g., volunteers and first responders), while some others such as TrygFonden Hjerteløber are used only by volunteers. It should be pointed out that companies responsible for maintaining technical aspects of the apps can have different names than the apps, for example both apps Sms-livräddare and TrygFonden Hjerteløber are managed by the company HeartRunner Sweden AB (2020).

While a few apps exist that are used in more than one country, in some countries such as Germany, many different initiatives have formed that utilize individual apps. This diversity in apps withing one country makes it difficult to implement country-wide standards and best practices. In addition, volunteers living in one region and working in another one might need to use two different apps if they can attend to emergencies in both regions.

## Managerial implications

To manage medical emergencies with limited resources and with the aim of upholding response time goals can become a complicated problem to solve. Depending on the country, policies and processes for the management of EMS are set by one organization for the entire country or by separate organizations for each federal state or even EMS region in that country (e.g., Germany). Usually these organizations have already established processes for the management of their services. Introducing new types of resources into the current layout of these systems will most likely require changes in their current processes. The proposed changes can then potentially be successful in reaching the implementation stage if (1) they are designed and modeled in a way that they will support the existing processes and (2) it is possible to show managers that their inclusion into the system can be a course of action against a shortage of resources in the emergency system and a help for meeting the response time targets. Otherwise, the output of research proposing changes will not be accepted by EMS staff or decision makers.

Changing processes, especially digitizing them, is often very challenging. Research areas addressing change management and digital transformation could offer valuable insights and methods to define and support necessary steps, such as digital transformation strategies (Matt et al. 2015). Focusing on healthcare, Kash et al. (2014) have listed success factors for strategic change management and Pferzinger and Rammerstorfer (2017) suggested the use of Design Thinking methods to support digital transformation in healthcare. Four states of change for transforming healthcare organizations have been proposed by Golden (2006).

The question of which subcategory of *human resources* or *equipment* to concentrate on for each *emergency type* is of high importance for EMS managers. They are responsible for setting out policies and plans that can improve the response to the emergencies. Therefore, selecting appropriate resources that can contribute best to a good response can play a vital role in response improvements. In this review, we have grouped new types of resources for managing daily medical emergencies to two categories (Sect. 3). Then, in Sects. 3.1 and 3.2 we have provided seven subcategories on both human resources and equipment and discussed them further in detail. This categorization can help EMS managers to find relevant references on particular topics of interest.

Consider volunteers among the subcategories of human resources as an example. Their involvement in the medical emergency responses include several aspects such as: how to alert volunteers and to which emergencies, what impact their involvement in the response would have, what dispatch method can be used, and how technology can support it both in terms of equipment and platforms (e.g., apps). Emergency managers will be able to find relevant research papers and existing apps and make decisions based on the findings of those research.

Type of emergency is another essential aspect that has high importance for emergency managers. Based on our categorization, emergency managers can find emergency-specific research papers. While most of the pre-EMS resources can be used in response to all emergency types, the use of some of them for certain emergency types might be more beneficial and reasonable. In some regions, the occurrence of particular emergency types can be more frequent than others. For example, in areas with high social turbulence with a history of shooting and knifing incidents, a need for pre-EMS services that can handle bleeding is more essential. Another example is the regions in which EMS services are weak and road traffic injuries and trauma leads to many casualties (e.g., some African countries). In such regions, laypersons and bystanders can play an effective role in strengthening pre-hospital efforts. As another example, in a region with an elderly population, the probability of a heart attack or cardiac arrest might be higher and pre-EMS services suitable for responses to OHCA are needed. Moreover, in such areas often the number of home-care personnel is higher than in other areas, and thus, these people could act as CFR and be the primary human resource in those regions. Therefore, interest of an emergency manager in different emergency types in relation to pre-EMS resources may be based on the work region and responsibilities. It is nevertheless worth mentioning that an analysis of actual demand and needs in a region should precede the integration of pre-EMS services.

## Directions for future research

Based on our findings from the review of 258 papers in this study, we make the following recommendations for future research. Our hope is that these recommendations can help researchers to address areas that have been less studied or need a different and new perspective. Consequently, over time, a body of work can form that represents a comprehensive and robust pre-EMS services literature. These recommendations can be categorized as those concerning *methodology*, *modes of transportation*, and *integration of services*. In the following, we present these recommendations in the order of these categories.

1. We found in our review that the majority of researchers, who have studied human resources, have used qualitative methods and methods that are common for the medical field. This leaves a noticeable gap for researchers of the OR domain to use different OR methods, such as optimization and simulation, to investigate the use of human resources in medical emergency responses.
2. Most of the researchers who have used optimization in their studies on any of the pre-EMS services, have developed a deterministic model. Although a deterministic setting makes the assumptions for modeling easier, it often makes the model less realistic. To depict the reality better and to deal with its associated uncertainties, researchers might want to consider the use of stochastic, probabilistic, or robust optimization as well as simulation-based optimization approaches.
3. In the existing body of works, a considerable number of studies are based on real-life experiments and simulations. However, not many researchers have used computer simulation as their methodology. Using computer simulation provides the opportunity to first test assumptions outside of the real world and without the use of real participants. Then, based on the successful settings of the computer simulation, a real-life experiment can be run. This can reduce the costs of experiments and potentially increase the chance of finding a successful setting. Simulation models have been successfully applied to planning problems within the classical EMS logistics, for example to analyze solutions of ambulance location models. Building simulation models for the use of AEDs and drones or incorporation of human resources in interventions in practice allows to analyze the potential impact and expected outcomes.
4. Existing studies on AED locations often consider that these devices are placed at fixed locations using different modeling techniques, such as coverage models, and people have to find them and get them to the scene of emergencies. However, one promising way of using these devices for emergency response is mobile AEDs. In this setup, AEDs can be delivered to the emergency scene by different modes of transport (e.g., drones or taxis). While our review shows that some research addresses the use of drones, further research seems promising and other modes of transport should also be investigated.
5. In the current literature on drones as part of pre-EMS services, researchers have focused mostly on technical and logistical perspectives, such as location planning or routing. We recommend researching and defining a more detailed blue print for the use of drones. This could include, for example, the use of drones for pickup and delivery of all kinds of medical equipment to the scene of emergencies, especially to areas that have less stationary equipment or to mountainous regions that are harder to reach. The benefits of using drones, potentially together with logistics and service design, is a research area widely open for further investigation.
6. Studies on the use of pre-EMS services for various types of emergencies is an area with potential for further research. For instance, the use of first responders or volunteers in response to trauma or bleeding emergencies, respectively, from both a theoretical and a practical point of view, could be investigated.
7. Overall, a comparison of different pre-EMS services and their designs and an analysis of their applicability to various practical settings is missing in the literature. For instance, a comparison of first responders systems, potentially together with different location approaches for AEDs, would be of interest.
8. As we found that no research has studied pre-EMS services in South American countries, this is also an area for further studies especially from practical perspectives. Studies related to the application of pre-EMS services in one country might be applicable in another country, with some necessary modifications to fit the system of the other country. However, this applicability needs to be investigated using real data from the secondary country (e.g., a South American country) or the use of computer simulation.
9. The introduction of pre-EMS services can have benefits for both the society and professionals. These benefits can mean a reduction of response times, for example, or potentially increasing survival chances of patients. The better pre-EMS services are integrated into the emergency response system, while maintaining their identity as additional resources, the higher the chance of success for the response system. Therefore, further research regarding the integration of these services into the current professional emergency response system can lead to major benefits, especially for practice.
10. To implement or expand pre-EMS services in practice, studies on the requirements and technical aspects of systems are needed, such as apps and their back and front ends, to help medical emergency managers decide between different services and actual implementations.
11. More than 20 independent apps for first responders and volunteers to attend OHCAs, for example, are used in several countries worldwide. Future research could provide best practices and standards for the design and use of these apps. In addition, OR approaches could be integrated in the design and implementation of these apps, for example to efficiently assign volunteers to OHCAs and to decide who to pick up an AED or whether an AED comes by drone.
12. As we have found out in the screening of our initial search results, publications on the use of pre-EMS services in disasters and large-scale emergencies already exist. However, studies covering the use of these services across the emergency spectrum including both ends, daily emergencies and disasters, and the design of more general models can be an interesting and valuable future research direction.

## Summary and conclusions

In this study, we have reviewed research on pre-EMS services that have been published in OR/OM or medical journals as well as conference proceedings until the end of the year 2020. We found a total of 258 papers published over a time span of 39 years (from 1982 to 2020). We focused our work on daily medical emergencies and presented an overview (macro-level analysis) of the existing literature in this field rather than giving a detailed (micro-level) analysis of a few individual papers. We hope that the output of this review will attract new researchers and provide valuable input and directions for future work to both researchers and medical emergency managers.

We categorized the papers based on the type of resource (i.e., human resources and equipment), type of emergency, type of data, and methodology. It is evident from the results of this review that researchers have mostly used qualitative methods to study human resources. While more researchers have used quantitative methods for equipment-related research, the overall focus on quantitative methods across all pre-EMS services has been relatively low. To test and validate the proposed hypotheses and models many researchers have used real data. So far, researchers have only considered the use of a single or very few resources for certain emergency types. Comparisons or studies on integrated use of several resources for multiple emergency types are still missing.
